# A call for further evidence regarding the association between metabolic syndrome, sepsis, and sepsis-related death^☆^

**DOI:** 10.1016/j.mmr.2026.100069

**Published:** 2026-09-29

**Authors:** Shamas Mohammed

**Affiliations:** Department of Plastic and Reconstructive Surgery, Royal Devon and Exeter Hospital, Royal Devon University Healthcare NHS Foundation Trust, Exeter EX2 5DW, United Kingdom

**Keywords:** Metabolic syndrome, Sepsis, UK Biobank, Kailuan Study

Metabolic syndrome (MetS) and its requisite conditions are diagnosed in a significant proportion of the worldwide population and are already known to increase the risk of several high-impact conditions, including cancer, diabetes, and cardiovascular disease [1]. However, there is limited evidence that provides a correlation between MetS, sepsis, and sepsis-related mortality. The study by Liu *et al.* [2] provided additional data through the analysis of two cohorts, the UK Biobank and the Kailuan Study, reporting statistically significant associations between MetS, sepsis, and sepsis-related mortality. In addition, Liu *et al.* [2] concluded that the more components of MetS a patient had, the greater the likelihood of developing sepsis, and the subsequent mortality associated with it.

This study adjusted for age, sex, educational level, assessment centre, smoking status, alcohol intake, physical activity level, and sleep deprivation for the Kailuan Study data [2]. The UK Biobank, however, provided significantly more information, including the Townsend Deprivation Index, diet, aspirin, and non-aspirin non-steroidal anti-inflammatory drug use, as well as nutritional supplementation [2]. Liu *et al.* [2] controlled for these risk factors by normalizing the data and providing hazard ratios adjusted by each risk factor, strengthening the relationship between MetS, sepsis, and sepsis-related mortality in their conclusion. They reported with hazard ratios that developing MetS was associated with worse outcomes, and that recovery from MetS improves outcomes, with appropriate sensitivity analysis to support this [2].

However, despite these strengths, there are several limitations of this study that warrant consideration and were not mentioned by the authors, particularly in relation to the Kailuan Study data [2]. This dataset relies primarily on the health records of 11 hospitals, from which 152,317 participants self-reported their sociodemographic and lifestyle factors. This dataset comprises, as acknowledged in the study, exclusively coal miners from this region. Whilst the cohort is homogeneous in occupation, this study does not delve into their specific roles at the mining company they worked in, and whether this would affect their lifestyle. Without explicit introduction in the study and due to physical activity data being self-reported, it is difficult to confirm if the paid physical labour was included in the analysis of physical activity, as had been done in other papers using the Kailuan Study cohort [3]. If there is incongruence between the participants’ true level and self-reported level of physical activity, it may explain why the level of physical activity showed no statistically significant associations with sepsis incidence (*P*=0.705) and sepsis-related mortality (*P*=0.729).

The outcome of sepsis-related mortality also warrants some consideration. The management of sepsis significantly alters the patient-level outcomes [4]. Additional statistical analysis, comparing outcomes across the 11 hospitals where patients received treatment, may improve the robustness of this study’s conclusions and support the assumption of consistency in sepsis management across hospitals. Alternatively, a statement in the method section stating that all hospitals adhered to the same sepsis guidelines may be sufficient.

Overall, Liu *et al.* [2] provided valuable insights into the relationships between MetS and sepsis after adjusting for a significant number of lifestyle covariates. The limitations stated are primarily with regards to the original chosen dataset. Further evidence from future studies adopting a rigorous, multi-centred design similar to that of Liu *et al.* [2] would corroborate their findings and enable these results to be generalized to a broader population.

## Abbreviations

MetS: Metabolic syndrome

## Ethics approval and consent to participate

Not applicable.

## Author contributions

SM was the sole author, including conceptualization, manuscript drafting. The author read and approved the final manuscript.

## Consent for publication

Not applicable.

## Funding

Not applicable.

## Competing interests

The author declares that there are no competing interests.

## Data Availability

All data associated with this study will be available upon reasonable request to the corresponding author. Primary data are available in the references below.
